# MicroRNA-29a-3p delivery via exosomes derived from engineered human mesenchymal stem cells exerts tumour suppressive effects by inhibiting migration and vasculogenic mimicry in glioma

**DOI:** 10.18632/aging.202424

**Published:** 2021-02-01

**Authors:** Zongpu Zhang, Xing Guo, Xiaofan Guo, Rui Yu, Mingyu Qian, Shaobo Wang, Xiao Gao, Wei Qiu, Qindong Guo, Jianye Xu, Zihang Chen, Huizhi Wang, Yanhua Qi, Rongrong Zhao, Hao Xue, Gang Li

**Affiliations:** 1 Department of Neurosurgery, Qilu Hospital, Cheeloo College of Medicine and Institute of Brain and Brain-Inspired Science, Shandong University, Jinan 250012, Shandong, China; 2 Shandong Key Laboratory of Brain Function Remodeling, Jinan 250012, Shandong, China; 3 Department of Neurosurgery, The Second Hospital of Shandong University, Jinan 250033, Shandong, China

**Keywords:** glioma, vasculogenic mimicry, mesenchymal stem cell, microRNA, exosome

## Abstract

Vasculogenic mimicry (VM), the formation of an alternative microvascular circulation independent of VEGF-driven angiogenesis, is reluctant to anti-angiogenesis therapy for glioma patients. However, treatments targeting VM are lacking due to the poor understanding of the molecular mechanism involved in VM formation. By analysing the TCGA database, microRNA-29a-3p (miR-29a-3p) was found to be highly expressed in normal brain tissue compared with glioma. An *in vitro* study revealed an inhibitory role for miR-29a-3p in glioma cell migration and VM formation, and further study confirmed that ROBO1 is a direct target of miR-29a-3p. Based on this, we engineered human mesenchymal stem cells (MSCs) to produce miR-29a-3p-overexpressing exosomes. Treatment with these exosomes attenuated migration and VM formation in glioma cells. Moreover, the anti-glioma role of miR-29a-3p and miR-29a-3p-overexpressing exosomes were confirmed *in vivo*. Overall, the present study demonstrates that MSCs can be used to produce miR-29a-3p-overexpressing exosomes, which have great potential for anti-VM therapy and may act as supplements to anti-angiogenetic therapy in the clinic.

## INTRODUCTION

Glioma is the most prevalent and deadly primary tumour of the central nervous system (CNS) in adults and it accounts for 80% of all primary brain tumours [1]. Vascular proliferation has been considered as one of the prominent features of gliomas which results in poor outcomes for glioma patients [2]. The anti-VEGF drug Avastin targets angiogenesis, which has been used for anti-glioma therapy [3]. However, patient outcomes are still poor, and for glioblastoma (GBM) patients, limited benefits are acquired despite the blocking of vascular endothelial cell-mediated angiogenesis [4]. Therefore, some salvage neovascularization independent of VEGF-induced angiogenesis in GBM must occur. Vasculogenic mimicry (VM) are the highly patterned vascular channels in tumour composed of a basement membrane stained positive with periodic acid-schiff (PAS) in the absence of endothelial cells and fibroblasts [5]. It has been reported that in glioma, VM structure is formed by transdifferentiated tumour cells [6]. The formation of VM bypasses the canonical pathways of angiogenesis and plays an important role in the maintenance of GBM malignancy [7].

VM formation is a consequence of hypoxia-induced epithelial-to-mesenchymal transition (EMT) in gliomas [8]. Studies have revealed that under hypoxic conditions, VM formation provides increased blood flow for oxygen supply and consequently promote the progression of GBM [8]. However, therapeutic methods targeting VM are still lacking due to poorly understanding for the molecular mechanisms involved in VM formation.

MicroRNAs (miRs) function as tumour suppressors or oncogenes [9]. We have previously found that several miRs are able to inhibit migration and VM formation in gliomas [10, 11]. However, the clinical use of these glioma suppressors is limited due to the lack of an ideal delivery system.

Exosomes are nanoparticles that are 30-100 nm in diameter [12]. Numerous biological molecules (including various miRs) are packaged into exosomes by donating cells and secreted into the extracellular microenvironments or the circulation [12]. Exosomes are accepted by target cells and subsequently alter the miR expression profile in target cells to induce variations in biological behaviour [12]. Recent research has demonstrated that miR-associated proteins were rarely found in exosomes, suggesting that exosomes do not contain major components of the miR biogenesis machinery [13]. Therefore, exosomes could serve as stable transferring vehicles for glioma suppressor miRs.

Mesenchymal stem cells (MSCs) are multipotent stromal cells that can differentiate into a variety of cell types, including osteoblasts, chondrocytes, myocytes and adipocytes [14]. Several studies have reported the delivery of tumour suppressive miRs to cancers via MSC-derived exosomes [15, 16]. Researches on glioma have revealed that exosomes show glioma-suppressive effect when antitumour-miRs were overexpressed in the exosome-donating MSCs [17–20], suggesting the considerable potential for the use of MSC-derived exosomes in glioma treatment. Despite the vital role of vascular proliferation in glioma progression, none of these studies focused primarily on anti-angiogenesis or anti-VM formation therapy for gliomas.

In the current study, we confirmed that miR-29a-3p attenuated migration and VM formation in glioma and thereby prolonged the survival time in xenografted nude mice model. Based on these findings, we verified that MSC-derived exosomes could be used as transferring vehicles for miR-29a-3p to decrease migration and VM formation both *in vitro* and *in vivo*. It may serve as an alternative treatment for glioma patients, especially for those who fail to respond to anti-VEGF therapy.

## RESULTS

### Glioma formed VM structures and showed aberrantly low expression level of miR-29a-3p

**Figure 1:**
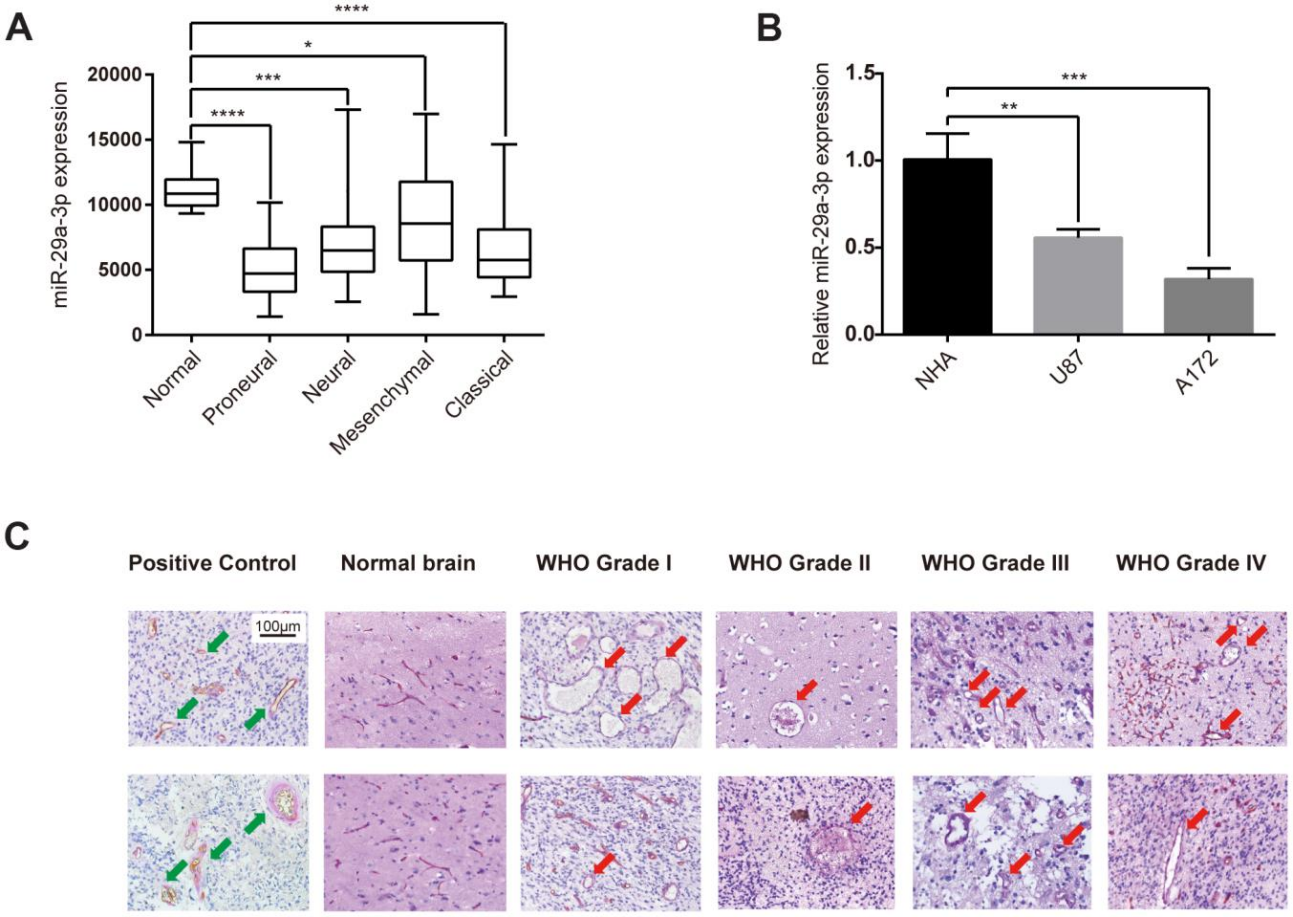
**Glioma formed VM structures and showed aberrantly low expression level of miR-29a-3p.** (**A**) Quantification of miR-29a-3p expression levels in gliomas of different subtypes in the TCGA database. Data are shown as the mean±SD, n=204, one-way ANOVA (*, P < 0.05). (**B**) miR-29a-3p expression in NHA, U87 and A172 cells. Data are shown as the mean±SD, n=3, one-way ANOVA (*, P < 0.05). (**C**) Representative images of CD34-PAS IHC staining of VM structures (red arrows; scale bar, 100 μm) in normal brain specimens (n=2) and in gliomas of different grades (n=45). Epithelium-lined vessels (CD34+/PAS+) were set as positive control (green arrows; scale bar, 100 μm).

Firstly, we studied the expression level of miR-29a-3p in normal brain tissue and in different subtypes of glioma in the TCGA database. As shown in the box plot, glioma tissue showed a significantly lower miR-29a-3p expression level compared with normal brain tissue (Figure 1A). These results were confirmed in cell lines. Compared with normal human astrocytes (NHA), the glioma cell lines U87 and A172 showed lower expression level of miR-29a-3p (Figure 1B). We then showed VM structures in glioma tissue by double staining for PAS and CD34. VM structures exhibit positive staining for PAS but negative for CD34, whereas the epithelium-lined vessels were CD34 positive (Figure 1C, Positive Control). No VM was found in the normal brain tissue, which suggested that VM structure is exclusively formed in glioma (Figure 1C). These results demonstrated that glioma formed VM structures and showed aberrantly low expression level of miR-29a-3p.

### miR-29a-3p inhibited migration and VM formation in glioma cells

**Figure 2:**
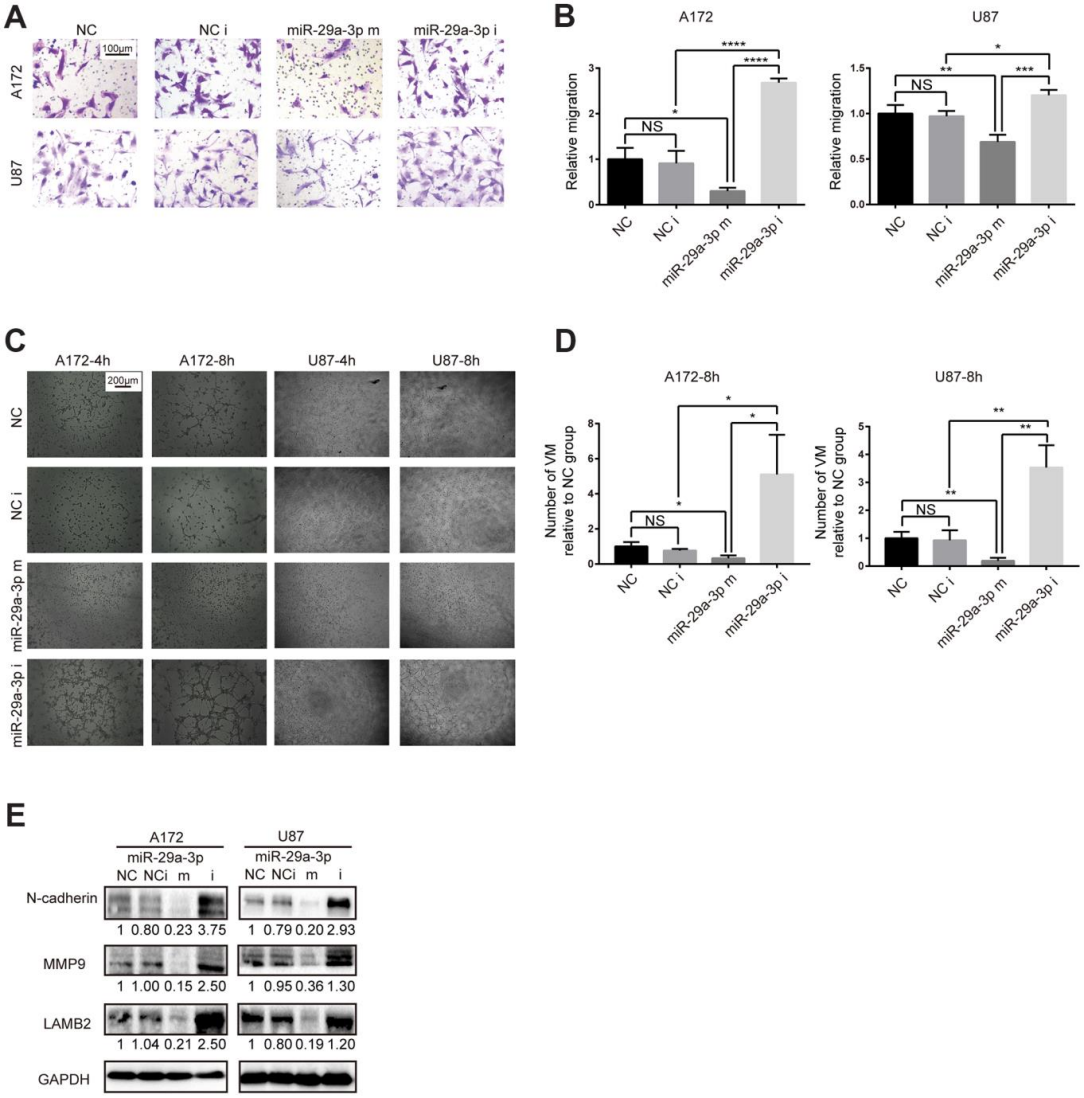
**miR-29a-3p inhibited migration and VM formation in glioma cells.** (**A**) The effect of miR-29a-3p on cell movement was assessed using transwell migration assays (scale bar, 100 μm; n=3). (**B**) Quantification of transwell migration assays in (**A**). Data are shown as the mean±SD, n=3, one-way ANOVA (*, P < 0.05). (**C**) Effect of miR-29a-3p on VM formation ability (scale bar, 200 μm; n=3). (**D**) Quantification of relative VM number in (**C**). Data are shown as the mean±SD, n=3, one-way ANOVA (*, P < 0.05). (**E**) Western blot analysis of protein levels of N-cadherin, MMP9 and LAMB2 in A172 and U87 cells. GAPDH was used as a whole-cell protein loading control. Results are from three independent experiments.

**Figure 3:**
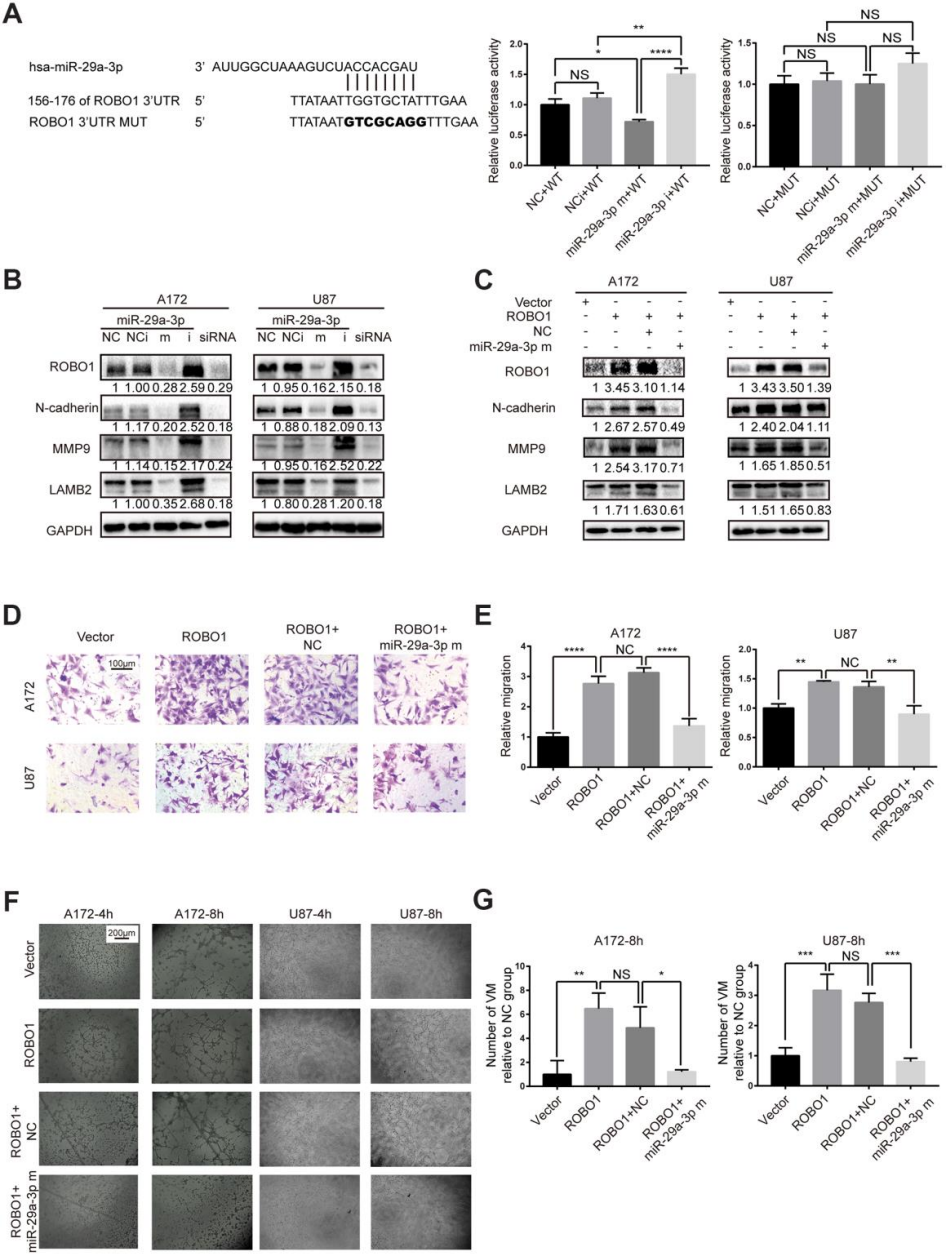
**miR-29a-3p inhibited migration and VM formation by directly targeting ROBO1.** (**A**) Schematic representation of the predicted binding sites for miR-29a-3p in the ROBO1 3′-UTR (wild type; WT) and the designed mutant versions (mutant; MUT) of the ROBO1 3’-UTR (left panel). Relative luciferase activity of HEK293T cells in the presence of the indicated treatments (middle and right plots). Data are shown as the mean±SD, n=3, one-way ANOVA (*, P < 0.05). (**B**) Western blot analysis of the protein level of ROBO1,N-cadherin, MMP9 and LAMB2 after miR-29a-3p transfection. Results are from three independent experiments. (**C**) Western blot analysis of the expression of ROBO1,N-cadherin, MMP9 and LAMB2 after ROBO1 and miR-29a-3p overexpression. Results are from three independent experiments. (**D**) Representative images for the transwell assay (scale bar, 100 μm; n=3). (**E**) Quantification of transwell migration assays in (**D**). Data are shown as the mean±SD, n=3, one-way ANOVA (*, P < 0.05). (**F**) Representative images for the VM formation assay (scale bar, 200 μm; n=3). (**G**) Quantification of relative VM numbers in (**F**). Data are shown as the mean±SD, n=3, one-way ANOVA (*, P < 0.05).

To explore whether the VM forming abilities in glioma was associated with lower expression of miR-29a-3p, we performed *in vitro* gain-of-function assays. Firstly, we examined the migration abilities in glioma cell lines after miR-29a-3p overexpression. Consistent with results in former studies [21], miR-29a-3p could inhibit migration in glioma (Figure 2A, 2B; NC: negative control; NC i: negative control inhibitor; miR-29a-3p m: miR-29a-3p mimics; miR-29a-3p i: miR-29a-3p inhibitors). It has been reported that migration plays an important role in VM formation [10]. We further explore whether miR-29a-3p had the ability to ameliorate VM formation as well. The results showed that four hours after being placed into the wells, the glioma cells began to exhibit different degrees of VM (Figure 2C). Overexpression of miR-29a-3p attenuated the formation of VM, while silencing miR-29a-3p promoted VM formation (Figure 2C, 2D). We then examined the molecular changes after miR-29a-3p transfection. Matrix metalloproteinase (MMP) 9 (MMP9) is an important factor associated with VM formation and migration [22] and LAMB2 is a VM-related protein [6]. We observed a decrease in MMP9 and LAMB2 protein level after miR-29a-3p overexpression, which indicated suppressed migration and VM formation pathways (Figure 3E). Considering that EMT contributes to VM formation and migration [8, 23], we detected the alteration of EMT-related protein after miR-29a-3p overexpression. We found that N-cadherin, the marker for EMT, was downregulated after miR-29a-3p overexpression, suggesting that miR-29a-3p inhibited the EMT process in glioma cells (Figure 3E). These results suggested that miR-29a-3p inhibited VM formation in glioma cells. In addition, migration and EMT, two processes closely associated to VM formation, were also impeded by miR-29a-3p.

### miR-29a-3p inhibited migration and VM formation by targeting ROBO1

To identify the target gene of miR-29a-3p, we used TargetScan and miRDB and found that the 3’ UTR of ROBO1 contains several highly conserved putative miR-29a-3p binding sites (Figure 3A, left panel). To verify that ROBO1 is a direct target of miR-29a-3p, 3’ UTR luciferase assays were performed (Figure 3A, middle and right panel). The results showed that overexpression of miR-29a-3p reduced the luciferase activity in wild type group (WT) but not in the mutant group (MUT), suggesting the direct binding of miR-29a-3p to the 3’UTR of ROBO1 (Figure 3A, middle and right panel). The expression level of ROBO1 was detected using western blotting (Figure 3B). The results showed that, following miR-29a-3p overexpression, ROBO1 was downregulated. Furthermore, ROBO1-knockdown decreased the protein levels of MMP9, LAMB2, and N-cadherin in a similar fashion as miR-29a-3p overexpression (Figure 3B). In contrast, overexpression of ROBO1 enhanced the expression of MMP9, LAMB2, and N-cadherin, indicating the pro-VM formation effects of ROBO1. However, these effects of ROBO1 could be offset by co-transfection with miR-29a-3p mimics (Figure 3C). To further confirm the opposing effects of ROBO1 and miR-29a-3p on migration and VM formation, we performed transwell assays (Figure 3D, 3E) and VM formation assays (Figure 3F, 3G). In accordance with the western blotting results, the overexpression of ROBO1 promoted migration (Figure 3D, 3E) and VM formation (Figure 3F, 3G). However, transfection of miR-29a-3p counteracted the effects of ROBO1 (Figure 3D, 3G). In summary, our results suggested that ROBO1 is a direct target for miR-29a-3p that mediates the anti-VM and anti-migration effects.

### Transfected MSCs transferred miR-29a-3p via exosomes and inhibited migration and VM formation in glioma

**Figure 4:**
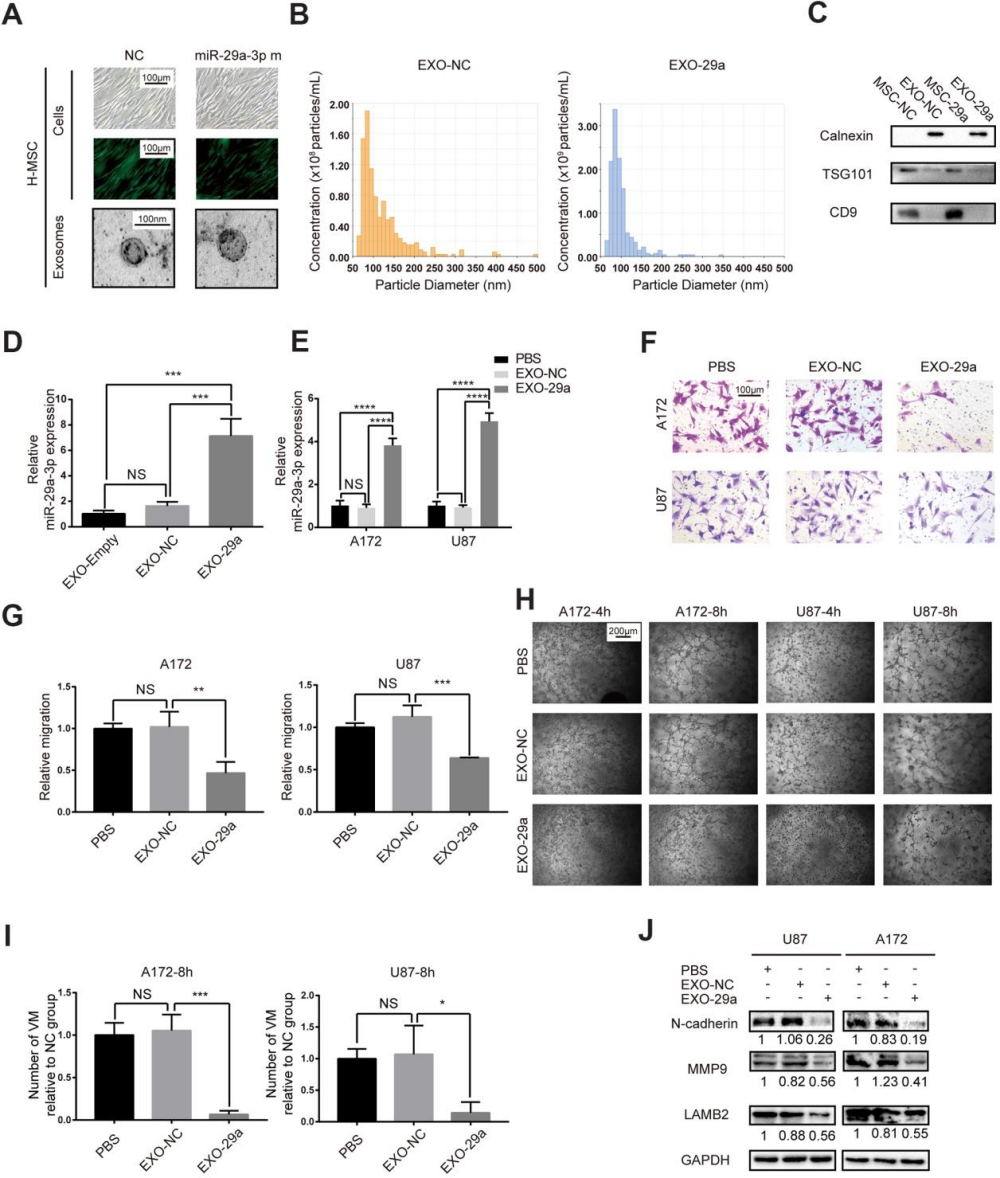
**Transfected MSCs transferred miR-29a-3p via exosomes and inhibited migration and VM formation in glioma.** (**A**) Representative images of human MSCs transfected with miR-29a-3p or an NC nucleotide sequence (scale bar, 100 μm) and the corresponding electron microscopic images of exosomes (scale bar=100 nm). (**B**) Nanoparticle tracking technology indicated an accumulation of particles of diameters of 50-100 nanometres. (**C**) Western blot analysis showing the presence of TSG101 and CD9 and the absence of calnexin in MSC-derived exosomes. Results are from three independent experiments. (**D**, **E**) PCR analysis of the miR-29a-3p level in exosomes (**D**) and glioma cell lines pretreated with exosomes (**E**). Data are shown as the mean±SD, n=3, one-way ANOVA (*, P < 0.05). (**F**) Migration capability detected by a transwell assay after treatment with miR-29a-3p-overexpressing exosomes (scale bar, 100 μm; n=3). (**G**) Quantification of transwell migration assays in (**F**). Data are shown as the mean±SD, n=3, one-way ANOVA (*, P < 0.05). (**H**) VM formation after treatment with miR-29a-3p-overexpressing exosomes (scale bar, 200 μm; n=3). (**I**) Quantification of relative VM numbers in (**H**). Data are shown as the mean±SD, n=3, one-way ANOVA (*, P < 0.05). (**J**) Protein levels of markers of migration and VM formation detected by western blotting after miR-29a-3p-overexpressing exosome (EXO-29a) treatment. Results are from three independent experiments.

Since we had proved the inhibitory role of miR-29a-3p in migration and VM formation, we speculated that artificially introducing miR-29a-3p into glioma may have a therapeutic potential. It has been reported that MSCs were able to package miRs into exosomes and transfer them to target cells, such as glioma cells [18]. We therefore overexpressed miR-29a-3p in human MSCs (H-MSC) using lentivirus (Figure 4A). Isolated MSCs-exosomes were characterized by TEM (for morphologic analysis; Figure 4A) and nanoparticle tracking technology (for size and concentration analysis; Figure 4B; EXO-NC: exosomes derived from miR-NC-transfected MSCs; EXO-29a: exosomes derived from miR-29a-3p-transfected MSCs). As expected, the particles exhibited spherical morphology with a diameter ranging from 50 to 100 nm (Figure 4A, 4B). We then performed western blotting to further confirm the presence of exosome markers TSG101 and CD9 but absence for endoplasmic membrane marker calnexin (Figure 4C). The upregulation of miR-29a-3p in MSC exosomes after transfection was confirmed by PCR, and the level of miR-29a-3p was increased 5-10-fold in the miR-29a-3p transfection group compared to that in the NC transfection group or untransfected group (Figure 4D, EXO-Empty: exosomes derived from untransfected MSCs). Furthermore, after treatment by EXO-29a, the expression level of miR-29a-3p in both glioma cell lines increased significantly, indicating the efficient transfer of miR-29a-3p via exosomes (Figure 4E). We then performed transwell assays for A172 and U87 glioma cells pre-treated with MSC exosomes for 48 h. The results indicated that EXO-29a significantly limited migration in both glioma cell lines (Figure 4F, 4G). Moreover, the VM formation capacity was ameliorated by EXO-29a (Figure 4H, 4I), which was consistent with the VM inhibitory capability of miR-29a-3p. Alterations in migration and VM related factors were observed (Figure 4J). EXO-29a could decrease N-cadherin, MMP9 and LAMB2 in the U87 and A172 cell lines (Figure 4J). Altogether, we verified the anti-VM and anti-migration capabilities of EXO-29a *in vitro*.

### *In vivo* study of miR-29a-3p and miR-29a-3p-transfected MSC exosomes

**Figure 5:**
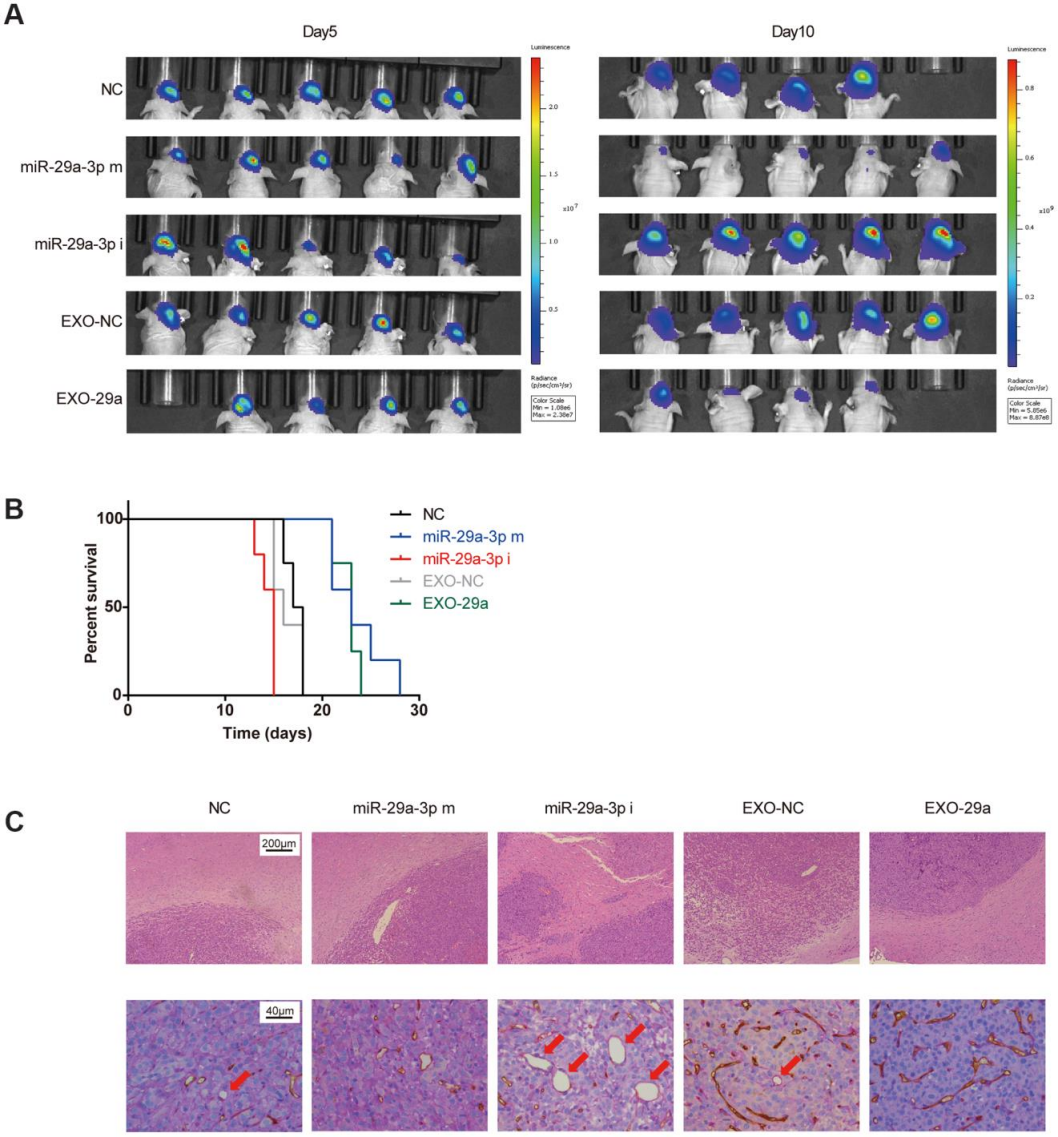
**Effects of exosomes overexpressing miR-29a-3p resembled the anti-glioma effects of miR-29a-3p overexpression *in vivo*.** (**A**) Bioluminescence imaging showed the tumour sizes on day 5 and day 10 after transplantation. (**B**) Kaplan–Meier survival curves for animals in different groups (miR-29a-3p m VS NC, p=0.0040; miR-29a-3p i VS NC, p=0.0074; EXO-29a VS NC, p=0.0091; EXO-29a VS EXO-NC, p=0.0060; EXO-NC VS NC, p=0.5433; log-rank test; n=23). (**C**) Representative images of the tumour/brain border with HE staining (upper panels; scale bar, 200 μm) and CD34-PAS IHC staining of VM structures (lower panels, red arrows; scale bar, 40 μm).

To extend our findings *in vivo*, we established an *in vivo* U87 xenograft nude mouse model. U87 glioma cells transfected with miR-NC, miR-29a-3p mimics or miR-29a-3p inhibitors were intracranially transplanted into nude mice (Figure 5A). Moreover, two more groups of nude mice were transplanted with U87 cells and administered with EXO-NC or EXO-29a four times per week after transplantation (Figure 5A). Five days after transplantation, the tumour volumes remained similar among the different groups, indicating that the initial numbers of injected cells were equal, whereas on day 10, the tumour burden of mice in the miR-29a-3p mimics group was much smaller than that in the miR-NC group or in the miR-29a-3p inhibitors group. It is worth noting that mice treated with EXO-29a showed a similar tumour burden as those in the miR-29a-3p mimic group, which indicated the therapeutic effect of EXO-29a *in vivo* (Figure 5A). Kaplan–Meier analysis showed that miR-29a-3p overexpression moderately prolonged the survival time (Figure 5B; median survival time: NC: 17.5 days; miR-29a-3p m: 23 days; miR-29a-3p i: 15 days). Treatment with EXO-29a also prolonged survival time compared with EXO-NC treatment or miR-NC transfection (Figure 5B; median survival time: NC: 17.5 days; EXO-NC: 16 days; EXO-29a: 23 days). To further determined the anti-migration effects *in vivo*, we conducted HE staining to show tumour/brain border in xenografted glioma tissues. The results indicated that there was less aggressive growth in gliomas overexpressing miR-29a-3p or treated with EXO-29a (Figure 5C, upper panels).

Furthermore, double staining for PAS and CD34 showed that miR-29a-3p overexpression and EXO-29a treatment hampered the VM formation abilities in xenograft gliomas. The VM structures could rarely be found in gliomas overexpressing miR-29a-3p or treated by EXO-29a (Figure 5C, lower panels).

In summary, we confirmed that miR-29a-3p inhibited migration and VM formation *in vivo*, which hampered the tumour growth and prolonged the survival of mice. Thereafter, administration of miR-29a-3p-transfected MSC exosomes was confirmed to have the anti-glioma capability *in vivo*.

## DISCUSSION

Glioma remains a fatal disease with poor outcomes. Grade IV gliomas (GBM) have the most aggressive clinical course (median survival between 14.5 and 16.6 months) [24, 25]. Anti-angiogenesis treatment is one of the therapeutic methodologies in addition to surgical resection [26]. However, the overall survival of glioma patients has failed to improve partially due to resistance to anti-angiogenesis therapy [4], suggesting that some methods of vessel formation independent of VEGF may exist in glioma. We have previously reported that VM, the formation of which is closely associated with the abnormal expression of several miRs (for example, miR-Let-7f and miR-584-3p), is correlated with poorer outcomes for glioma patients [10, 11]. Due to the absence of endothelial cells, VM structures were resistant to anti-angiogenesis agents targeting endothelial cells [6]. Moreover, it has been reported that the anti-VEGF drug Avastin increases VM in glioma [6]. Altogether, it is essential to explore a treatment targeting VM as a supplement for anti-angiogenesis therapy.

In this study, our results reveal that miR-29a-3p, which is a tumour suppressor in various malignant tumours, attenuated VM formation in gliomas. ROBO1 was a direct target for miR-29a-3p and knockdown of ROBO1 could yield the anti-VM effect in a same fashion. VM is closely associated with migration and EMT [8, 23]. Ling et al. reported that VM formation in glioma is probably attributed to EMT-induced cell plasticity elevation and MMP-induced extracellular matrix remodelling [23]. The administration of EMT blocker SB203580 decreases N-cadherin expression and impairs VM formation abilities in glioma [23]. The downregulation of MMP9, a well-defined migration marker, diminishes the VM formation ability as well [22]. Furthermore, EMT induced migration in tumour cells whereas MMP9 is a potential EMT-promoting factor [27]. Therefore, we also confirmed the inhibitory role of miR-29a-3p in migration. N-cadherin, MMP9 and LAMB2 (the markers for EMT, migration and VM, respectively) were systematically inhibited by miR-29a-3p.

MSCs can be isolated from various tissues, including bone marrow, umbilical cord, and adipose tissue [28, 29]. Recently, the technologies for MSC ex vivo culture and engineering have been developed [28, 29]. Some researchers have utilized MSCs for anti-tumour agents delivery [30, 31]. However, direct intravenous administration may trap MSCs inside the lungs due to the large size of MSCs. Injection MSCs into tumour nourishing arteries may prevent this but would drastically increase the complexity of the treatment [32]. As a result, MSC-derived exosomes may be an alternative method to deliver anti-tumour agents.

MSCs have the strong capability to secrete exosomes [33] and various miRs are packaged into exosomes by MSCs [19, 34]. These findings imply the potential delivery of miRs with engineered MSC exosomes. In the current study, we transfected human MSCs with miR-29a-3p. We observed an elevation of miR-29a-3p in the MSCs exosomes. Consistent with the anti-glioma effects of miR-29a-3p, the miR-29a-3p overexpressing exosomes attenuated the migration and VM formation *in vitro* and *in vivo*.

Due to time constraints, we did not explore the mechanism by which ROBO1 inhibits VM formation. It has been reported that VMs are probably originated from glioma stem cells [35–37]; hence, it is probable that ROBO1 abolishes VM formation ability by suppressing stemness. If so, exosomes derived from miR-29a-3p-overexpressing MSCs would have even broader application. Since glioma stem cells are associated with resistance to radiotherapy and chemotherapy [38, 39], the administration of engineered MSC-derived exosomes may reduce resistance to radiotherapy, chemotherapy and antiangiogenesis therapy.

In summary, we revealed the inhibiting role of miR-29a-3p on migration and VM formation in glioma. To utilize this anti-tumor effects of miR-29a-3p, we modified MSCs as a “bio-factory” for exosomes overexpressing miR-29a-3p. EXO-29a attenuated migration and VM formation and hence inhibited the growth of glioma. It is promising to use EXO-29a as a supplement for anti-VEGF therapy which would synergistically decrease the blood supply in glioma. Furthermore, anti-VEGF drugs could promote the formation of VM in glioma [6]; to prevent this side effect, inhibiting VM may be considered as an integral part of antiangiogenesis therapy in the future.

## MATERIALS AND METHODS

### The cancer genome atlas (TCGA) databases and clinical specimens

TCGA Research Network data for microRNA expression microarrays and associated clinical information for samples were downloaded from Betastasis.com (http://www.betastasis.com/). The data were analysed with GraphPad Prism. Glioma tissues (WHO I-IV, n=45) and normal brain tissue (n=2) embedded in paraffin had been collected from patients who underwent surgery at the Department of Neurosurgery at Qilu Hospital of Shandong University. Normal brain tissue samples were collected from severe traumatic brain injury patients who underwent partial resection of the normal brain.

### Immunohistochemistry (IHC)

Sections were obtained from paraffin-embedded tissues from normal brains and human gliomas of different grades. The sections were heated, deparaffinized, rehydrated and placed in sodium citrate buffer (pH 6.0) for antigen retrieval, and the endogenous peroxidase activity was blocked with 3% hydrogen peroxide. The slides were blocked with 10% normal goat serum and incubated with primary antibody (rabbit anti-CD34 monoclonal antibody, 1:500 dilution, ab81289; Abcam; UK) at 4° C overnight. The images were visualized by following standard protocols using a horseradish-peroxidase-conjugated secondary antibody and 3,3′-diaminobenzidine (DAB) as a substrate, and PAS staining was used to visualize the matrix-associated vascular channels. Sections were incubated with normal rabbit serum to generate the negative controls. The slides were counterstained with haematoxylin, and typical images were obtained using a Leica DM 2500 microscope.

### Cell culture

Human glioma cell lines (U87MG and A172) and normal human astrocytes (NHAs) were obtained from the Culture Collection of the Chinese Academy of Sciences (Shanghai, China) and cultured in Dulbecco’s modified Eagle medium (DMEM; Thermo Fisher Scientific; USA) with 10% fetal bovine serum (FBS; Gibco; USA). Human bone marrow-derived MSCs were purchased from Cyagen (Suzhou, China) and cultured in mesenchymal stem cell complete medium (HUXMA-90011; Cyagen; China). These cell lines were maintained in a humidified chamber containing 5% CO2 at 37° C.

### MiR-29a-3p/ROBO1 overexpression and knockdown

Small interfering RNA (siRNA) for ROBO1, ROBO1-overexpressing vector and empty vector were purchased from Genepharma (Shanghai, China). Lentiviruses encoding miR-29a-3p mimics (miR-29a-3p m), miR-29a-3p inhibitor (miR-29a-3p i), negative control (NC) and inhibitor-negative control (NC i) were purchased from Genechem (Shanghai, China). Transient cell transfections (siRNA and vector) were performed using Lipofectamine^TM^ 3000 reagent (Thermo Fisher Scientific; USA) according to the manufacturer’s protocol. Transfections with lentivirus were performed with a MOI=10 in the two glioma cell lines and a MOI=10 in the human MSCs.

### Exosome isolation and identification

Exosomes from MSCs were isolated from conditioned medium from MSCs, and the procedures used for isolation were performed as previously described [40]. The exosomes were stored at −80° C and verified by electron microscopy and nanoparticle tracking technology (Nanosight™).

### VM formation assay

Ninety-six-well tissue culture plates were coated with Matrigel (50 μl/well; BD Biosciences; France), and U87 and A172 cells were seeded in the wells (3x10^4^ cells/well) and cultured in FBS-free medium (100 μl/well). Images were captured at 4 h and 8 h using an inverted Olympus microscope (DP72, Japan). The number of tubules was analyzed using ImageJ software [41].

### Transwell migration assays

Glioma cells were added to the top chamber in serum-free media. The bottom chamber was filled with DMEM containing 10% FBS. After 6 h (U87) or 13 h (A172) of incubation, the cells in the top chamber were removed using a cotton swab, and the membrane was fixed in 4% paraformaldehyde for 15 min and stained with crystal violet for 15 min. Three fields of adherent cells from each well were photographed randomly.

### Luciferase reporter assay

The reporter genes containing pGL3-ROBO1 and pGL3-mutROBO1 were synthesized by Bio-Asia (Jinan, China). The 293T cells were co-transfected with the luciferase reporters and the miR-29a-3p mimics/inhibitors. 48 h later, the activity of the reporter protein was measured using a luciferase assay kit (Promega; USA) according to the manufacturer’s instructions.

### Western blotting

The harvested cells were lysed using heat denaturation in RIPA cell lysis buffer. The protein lysates were loaded and separated using SDS-PAGE and then transferred to a polyvinylidene difluoride (PVDF) membrane. The blots were incubated with primary antibodies against ROBO1 (rabbit anti-ROBO1 polyclonal antibody, 1:500 dilution, 20219-1-AP; Proteintech; China), LAMB2 (rabbit anti-LAMB2 polyclonal antibody, 1:1000 dilution, 10895-1-AP; Proteintech; China), N-cadherin, MMP9, and GAPDH (1:1000; Cell Signaling Technology; USA). To visualize the protein bands, enhanced chemiluminescence (ECL, Millipore, Bedford, USA) was used. The intensity of the protein bands was analysed using ImageJ software and normalized to GAPDH.

### Quantitative real-time PCR (qRT-PCR)

Total RNA was isolated from glioma cells using Trizol reagent (Invitrogen, Life Technologies). Reverse transcription was performed using 2 μg of total RNA and the High Capacity cDNA Reverse Transcription Kit (Toyobo, FSQ-101) according to the manufacturer’s protocol. The cDNA was subject to real-time PCR using the Mx-3000P Quantitative PCR System (Stratagene). The primers for miR-29a-3p were 5-CATCTGACTAGCACCATCTGAAAT-3 and 5-TATGGTTTTGACGACTGTGTGAT-3. The primers for U6 were 5-CAGCACATATACTAAAATTGGAACG-3 and 5-ACGAATTTGCGTGTCATCC-3. The relative miR expression was normalized to that of U6.

### Intracranial mouse model

To establish the intracranial gliomas, U87MG luciferase cells (5×10^5^) transfected with lenti-miR-29a-3p, lenti-miR-29a-3p-inhibitors or lenti-control virus were stereotactically implanted into the brains of 4-week-old nude mice (SLAC Laboratory Animal Center, Shanghai, China). For the MSC exosome treatment experiment, U87MG luciferase cells (5×10^5^) were implanted, and the mice were intravenously injected with exosomes (100 μg/mouse) four times per week for 3 weeks. Bioluminescence imaging was used to detect intracranial tumour growth. Kaplan-Meier survival curves were plotted to determine the survival time. When the mice were moribund or cachectic, the tumour tissues were harvested, fixed in formalin, embedded in paraffin, cut into sections and HE stained.

### Statistical analysis

Data analysis was performed and visualized using GraphPad Prism. Each experiment was carried out at least in triplicate, and all results are presented as the means±SD. One-way ANOVA test was used to assess statistical significance. Kaplan–Meier survival curves were also constructed, and log-rank tests in GraphPad Prism software were used to assess survival. The data were considered significant with the following P values: P value < 0.05, denoted by “*”; P value < 0.01, denoted by “**”; P value < 0.001, denoted by “***”; and P value < 0.0001, denoted by “****.” P values > 0.05 were considered not significant and are denoted by “ns”.
